# Diets of Men and Women in Rural Bangladesh Are Equitable but Suboptimal

**DOI:** 10.1016/j.cdnut.2023.100107

**Published:** 2023-06-02

**Authors:** Fiona M. Coleman, Akhter U. Ahmed, Agnes R. Quisumbing, Shalini Roy, John Hoddinott

**Affiliations:** 1 Division of Nutritional Sciences, Cornell University, Ithaca, NY; 2 Poverty, Health, and Nutrition Division, International Food Policy Research Institute, Washington, DC; 3 Charles H. Dyson School of Applied Economics and Management, Cornell University, Ithaca, NY

**Keywords:** intrahousehold food allocation, Bangladesh, equity, gender, diet quality

## Abstract

**Background:**

Recent evidence suggests that diet inequities between men and women may have diminished within rural Bangladeshi households. However, this has not been directly tested with appropriate physiologic adjustments and it is unclear whether changes have occurred across socioeconomic strata. Understanding intrahousehold dietary patterns at different points on the income and food-security distribution in rural Bangladesh—particularly, within ultrapoor and farm households—is important for appropriate design of gender-sensitive and nutrition-sensitive interventions, which often target these groups.

**Objective:**

Using 2012 and 2016 data, we aimed to examine gender differences in diet quantity and quality among ultrapoor and farm households in rural Bangladesh.

**Methods:**

The study used baseline 24-h dietary data from 2 randomized control trials conducted in rural Bangladesh: the Transfer Modality Research Initiative (ultrapoor households) and the Agriculture, Nutrition, and Gender Linkages project (farm households). Ordinary least squares regressions with household-level fixed effects tested for gender differences among constructed diet measures, such as caloric intake, caloric adequacy ratio, dietary diversity score, global diet quality score, and probability of consuming moderate or high levels of healthy food groups.

**Results:**

In both samples, on average, women consumed fewer calories than men in the same households but consumed near equal or more in reference to their caloric needs. Women scored <1% lower than men on diet quality indicators and showed similar probabilities to men of consuming healthy foods. Most men and women in both samples were calorically inadequate (>60%) and recorded poor diet quality scores that indicated high risk of nutrient inadequacy and chronic disease (>95%).

**Conclusions:**

In both ultrapoor and farm households, although men record higher intake quantities and diet quality scores, the apparent male advantage disappear when energy requirements and the magnitudes of difference are considered. Diets of men and women in these rural Bangladeshi households are equitable but suboptimal.

## Introduction

In the early 1980s, seminal work by Chen et al. (1981) and Kynch and Sen (1983) brought attention to the abnormally high female to male mortality ratios in China, South Asia, and West Asia: a phenomenon described as “missing women.” In addition, others posited that a primary determinant of excess female mortality was gender-based disparities in nutrient consumption within households [[1], [2], [3], [4], [5]]. Over the next 15 y, a series of studies on household food allocation in Bangladesh, Nepal, and India provided confirmatory evidence that girls and women were disadvantaged in their access to food—in both energy intake and dietary quality [3,[6], [7], [8]]. Although a few studies suggested that at least part of these observed differences in food intake were explained by male–female differences in energy needs [9,10], the dominant narrative that emerged was, quoting Harris-Fry et al. [8], “In South Asia, the scant evidence available suggests that women, are discriminated against and receive less than their ‘fair share’, particularly in the allocation of high status, nutrient-rich food.”

Although low caloric and nutrient intake reflect, partly, low household income and food insecurity, patriarchal norms were seen as the key explanation for low intake of nutrients by females relative to that by males. This argument has been particularly prominent in the Bangladesh and South Asia context, where women have historically been subservient to men and encouraged to make sacrifices in favor of other household members, particularly in times of food scarcity [[6], [7], [8]]. Writing more than 40 y ago, Rizvi [7] noted that in Bangladesh, “From the time of puberty, a girl is expected to be a near perfect approximation of an idealized wife and mother. It is from this time that a female becomes least demanding and consequently receives a smaller allocation of food. […] while this custom of considering women as the epitome of sacrifice is emphasized in all socioeconomic groups, adherence is strongest in the low-income group where a limited supply of food leads to greater inequality.”

Given the established literature, few have reexamined the intrahousehold allocation of food in Bangladesh in the past 15 y, exceptions being the studies by D’Souza and Tandon [11] and Brown et al. [12], both of which conclude that a promale bias in household food allocation remains. However, in the study by D’Souza and Tandon [11], the magnitudes of gender differences in nutrient intake were small (<2% differences in caloric and protein shortfalls between male heads and their spouses) and relatively uncommon (differences were conditional on being undernourished, but most of the sample was considered adequately nourished) [11]. Moreover, although Brown et al. [12] claim that men consumed inequitably larger shares of household food than women and other household members, a closer inspection of their results reveals that women fared better than men when intakes (calories, protein, and food budget shares) were adjusted for sex-specific and age group–specific requirements. Furthermore, neither study explicitly adjusts for physical activity level (PAL) in their nutritional outcome comparisons, which, if considered, would likely further decrease any male advantage.

In the past 2 decades, Bangladesh has experienced remarkable growth. Since 2000, incomes have increased 6-fold, the prevalence of extreme poverty has been halved, and per capita caloric and protein supply has risen yearly [13,14]. Similarly, diets have significantly diversified in rural Bangladesh in the past decade for men and women alike, although the intake of carbohydrates is exceptionally high relative to other macronutrients [15]. Indicators of female opportunities and health have trended upward as well, with females surpassing males in some areas. For example, school enrollment and literacy rates are higher for females, women’s economic participation has increased, and the female mortality rate has declined to the point where is now lower than that of males [13]—changes that indicate an improvement in the status and conditions of women in Bangladesh.

These dramatic changes warrant a reexamination of the extent to which women in Bangladesh are discriminated against in the allocation of household food, regarding not just the quantity of food intake but also the overall healthfulness of diet. We do so in this study, making use of 2 new data sources that have become available since 2012 that represent 2 subpopulations of rural Bangladesh: 1) ultrapoor households and 2) mostly land-owning, food-secure farm households (referred to as “farm households” hereafter) [8].

We chose these samples to study gender bias in food allocation for 2 reasons. First, previous research has indicated that allocation patterns vary among different socioeconomic groups, with poorer and more food-insecure households experiencing more pronounced disparities in overall intake and less food-scarce, higher socioeconomic households experiencing less inequity in the distribution of staple foods but more inequity with more desirable nutrient-rich foods [8]. Thus, it is important to examine gender inequities in diets within different socioeconomic groups, which are represented in these samples of ultrapoor and farm households. Second, many large-scale development programs such as social protection and agriculture interventions are focused on ultrapoor households and farm households, respectively, and there is growing interest in making these types of interventions more gender and nutrition sensitive—particularly for households with nutritionally vulnerable household members, such as pregnant and lactating women and children younger than 2 y. Accordingly, for appropriate program design, it is important to understand the intrahousehold dietary patterns for these types of households. However, to do so requires having large enough samples of households with these socioeconomic and demographic characteristics and dietary intake information from all household members, which is uncommon in large-scale nutrition surveys that are often not powered to study subpopulation nuances and typically collect dietary information at the household level instead of the individual level. Fortunately, these 2 data sets include detailed food intake data from all individuals within 4992 ultrapoor households and 4000 farm households, allowing us to closely study intrahousehold allocation within these interest groups.

This study is closely related to that by Ahmed et al. [15] who used a nationally representative data set of rural Bangladesh to examine dietary trends over age, gender, and income groups between 2011 and 2018. Although their study focused on between-year and between-income group comparisons—finding that diet diversity has improved over time for all gender and age groups but significant disparities by household income persist among those groups—their results suggest that gender inequality in diets has diminished in recent years; however, gender differences were not directly tested. This study builds on this work by assessing whether diets significantly differ between men and women in rural Bangladeshi households of varying socioeconomic status.

The aim of this study is to determine whether women in ultrapoor and farm households in rural Bangladesh are disproportionally disadvantaged in their quantity and quality of diets. [The terms “male and female” and “men and women” are used interchangeably in this study to refer to gender. Typically, we use “male and female” when comparing outcomes that include younger age ranges (e.g., children or young adolescents) and “men and women” when discussing outcomes for adults and older adolescents (individuals ages 15 y or older). In Bangladesh, one can argue that the influence of gender roles (socially determined) affects allocations to males and females across the age distribution.] In doing so, we consider occupation and sex-based differences in energy requirements and assess diet quality with various measures, including the recently developed GDQS [16].

## Methods

### Study settings

We used baseline survey data from 2 cluster-randomized control trials conducted in rural Bangladesh: the Transfer Modality Research Initiative (TMRI; ultrapoor household sample) and the Agriculture, Nutrition, and Gender Linkages (ANGeL) project (farm-household sample). The baseline survey for TMRI was performed in 2012 in 2 regions of rural Bangladesh: the Rangpur division (province) in the northwest and Barisal and Khulna divisions in the south. The baseline survey for ANGeL was conducted in 2015–2016 in all 8 divisions of Bangladesh.

### Sample designs

Study participants in TMRI were selected using a multistage sampling process that was performed separately for the northwest and south regions. First, 5 upazilas (subdistricts) from each region were randomly selected from a list of upazilas with high poverty rates (i.e., the proportion of households living under the extreme poverty line was ≥33% in 2010). From the sample of upazilas in each region, a list comprising all villages was prepared. From the list, 250 villages per region were randomly selected, and a complete census was conducted in each village. Then, the census data were used to randomly select 10 households within each village that met the following criteria: they were poor (based on a set of poverty indicators described in the study by Ahmed et al. [16]), had ≥1 child aged between 0 and 24 mo, and were not receiving any payments from a government social protection program. The total sample in the 2 regions included 500 villages and 5000 households.

The ANGeL study included a sample of 160 agricultural “blocks” in 16 rural upazilas (subdistricts). These were localities that were deemed to be agroecologically suitable for crop diversification and had good market connectivity. These upazilas belong to 16 districts in 8 divisions of Bangladesh. From this sample, random sampling was conducted as follows: 10 agricultural blocks were selected at random from each subdistrict (yielding 160 blocks), and one village from each block was randomly selected. Within each of these villages, 25 farm households with ≥1 child aged between 0 and 24 mo (index child) were randomly selected to participate. This yielded a sample of 4000 households.

### Data collection

The same data collection processes were used for both the TMRI and ANGeL trials. In-person interviews were performed by enumerators from a Bangladeshi research firm, Data Analysis and Technical Assistance (DATA), in March 2012 for TMRI and from November 2015 to January 2016 for ANGeL (baseline survey for each trial). The total baseline survey sample for TMRI was 4992 households (of the 5,000 households in the TMRI trial, 8 households could not be interviewed) that contained 25,804 individuals (*n* = 12,270 adults aged 19 y or older; *n* = 700 adolescents aged 15–18 y). The ANGeL baseline survey included 4000 households containing 20,373 individuals (*n* = 11,913 adults aged 19 y or older; *n* = 941 adolescents aged 15–18 y).

In each household, a questionnaire was separately administered to men and women. Household heads (typically men) were interviewed on household demographic and socioeconomic characteristics, such as the gender of household members, livelihood activities of all adult household members (occupations and hours spent in each occupation), years of education completed and literacy level of each household member, household food purchases and consumption during the last 7 d [7-d dietary recall (7DDR)], and wealth indicators such as land ownership, assets, and household expenditures. The mother of the index child was interviewed on food preparation and sharing practices during the last 24 h and household food-security status. All women were asked about their lactation and pregnancy status. For the ANGeL surveys, height and weight were also collected for all household members.

Individual-level dietary intake data were collected using a combination of single-day 24-h dietary recalls (24HRs) and food-weighing methods. In each household, the person primarily in charge of preparing and serving meals (in nearly all cases, the female spouse of the male household head) was interviewed regarding the foods consumed (within and outside the home) the previous day by all household members. If the previous day was special (e.g., the household ate special foods, ate more or less than usual, or did not eat because they were fasting), the respondent was asked to describe the foods consumed 2 d before or the last “normal” day. For each dish consumed, information was collected on the ingredients, the raw weight of ingredients, and the cooked weight of the dish. Then, the respondent was asked about the portion size (grams) consumed by each household member. Caloric content was estimated by matching food items and mixed dishes to a food composition table specific to Bangladesh [17,18]. The TMRI and ANGeL 24HR dietary recall data were collected and processed using the same methods.

### Outcome variables

Using the 24HR food intake data and other relevant information such as age, occupation, and lactation and pregnancy status, we constructed the following 6 measures of diet for all individuals aged 15 y and older:

(1) Caloric intake from the previous day was used to compare the overall intake quantity between household members.
(2) Caloric adequacy ratio (CAR), the ratio of caloric intake to estimated energy requirements (EER) for calories, was used to determine the caloric adequacy of diets. A CAR value of 1 represents a calorically adequate diet as follows:

$$Caloric\;Adequacy\;Ratio=\frac{caloric\;intake}{EER}$$

Energy requirements were estimated using the FAO guidelines and a table of Bangladeshi-specific requirements developed by Waid et al. [19,20]. Adult requirements (19 y or older) were specific to an individual’s PAL [as determined by one’s self-reported primary occupation (Table A17, Appendix, Section 5) for PAL categories by occupation and gender], pregnancy status (including months of pregnancy for the TMRI sample), lactation status (based on the age of the breastfed child), and the ideal adult weight for each age and sex group (based on the mean heights of Bangladeshi adults) [[19], [20], [21]]. EERs for adolescents 15–18 y were based on the World Health Organization (WHO) growth reference for BMI for age and height for age, with adjustments for the short stature of Bangladeshis (see Waid et al. [20] for details). Adolescent values were further adjusted for occupation-related PALs and pregnancy or lactation status if applicable. In a supplemental analysis, we included children as well (**Appendix, Section 3**). EERs for children aged 2–9 y were estimated using WHO growth standards and growth reference curves, whereas EERs for children aged 10–15 y were estimated using the same method as adolescents aged 15–18 y [20,21]. All children were assumed to perform a moderate PAL level. See Tables A13–A15 for EER values by age, sex, and PAL group and for pregnancy and lactation adjustments.

(3) Dietary diversity score (DDS): We constructed our measure of DDS using the following food group categories: grains, white roots, and tubers; pulses (beans, peas, and lentils); nuts and seeds; dairy; meat, poultry, and fish; eggs; dark green leafy vegetables; other vitamin A–rich fruits and vegetables; other vegetables; and other fruits. The DDS ranges from 0 to 10, where a score of 5 (consumption of 5 of the 10 groups) generally indicates a minimally adequate diverse diet, although this cutoff has only been validated as a proxy for micronutrient adequacy in nonpregnant, nonlactating (NPNL) women aged 15–49 y [22].
(4) The global diet quality score (GDQS), which captures dimensions of diet quality related to both undernutrition and overnutrition and their associated risks for adults (aged 14 y or older) [23], was used to assess overall diet healthfulness. This measure was used in addition to the DDS because it allows for a more granular inspection of diets and considers the consumption of unhealthy foods. Based on the methods described in GDQS Tabulation Guidelines [24], we allocated the 24HR food intake data to 25 food groups: 16 healthy (citrus fruits, deep orange fruits, other fruits, dark green leafy vegetables, cruciferous vegetables, deep orange vegetables, other vegetables, legumes, deep orange tubers, nuts and seeds, whole grains, liquid oils, fish and shellfish, poultry and game meat, low-fat dairy, and eggs); 7 unhealthy (processed meats, refined grains and baked goods, sugar-sweetened beverages, sweets and ice cream, juices, white roots and tubers, and purchased deep-fried foods); and 2 groups (red meat and high-fat dairy) that are healthy when consumed at a moderate level but unhealthy when consumed in excessive amounts. Higher consumption of healthy foods increases the score, whereas consumption of unhealthy foods decreases the score. The GDQS ranges from 0 to 49. A score of ≥23 is considered a low risk of nutrient inadequacy and chronic disease; a score of 15–22 and <15 was associated with a moderate and high risk of these outcomes, respectively [23,24]. See Table A16 for further details on the GDQS scoring method.
(5,6) To determine whether differences in dietary quality scores reflected appreciable consumption disparities across food groups, we estimated the following for each respondent: i) mean probability of consuming moderate or high levels of all healthy food groups and ii) probability of consuming a moderate or high level of each healthy food group. For each of the 18 “healthy” GDQS food groups (including the 2 groups that are healthy when consumed at a moderate level), we created a dichotomous variable that took the value of 1 if an individual had a moderate or high consumption of the food group and 0 if an individual did not consume the food group or if consumption was low. The consumption levels were based on the food group–specific gram-per-day cutoffs used for scoring the GDQS groups (see Table A16 for GDQS gram-per-day cutoffs).

### Statistical analysis

Statistical analyses were performed using Stata 16.1 (StataCorp LP). The primary analyses included only adolescents and adults (aged 15 y and older) and were conducted separately for the ultrapoor household (TMRI baseline data) and farm-household samples (ANGeL baseline data).

First, we characterized and compared the samples by describing the households [number of household members, gender of household head, land ownership, and food-security status as measured by food consumption score (FCS)] and individuals [age, education level, literacy, PAL, occupation, relation to household head, pregnancy and lactation status, and BMI (farm-household sample only)]. Next, we compared unadjusted dietary indicator values between men and women (caloric intake, CAR, DDS, GDQS, and the mean probability of consuming moderate or high levels of all healthy foods) and compared the distributions of dietary scores (CAR, DDS, and GDQS) with cutoff values that indicate a high risk for poor nutritional outcomes. We tested for gender differences across select individual characteristics and unadjusted dietary outcomes using paired *t* tests for continuous variables and χ^2^ tests for categorical and dichotomous variables.

Subsequently, we used ordinary least squares (OLS) regressions to test for gender differences in dietary indicators with adjustments for individual and between-household factors. We adjusted for the age of the individual (log transformed to limit the influence of outliers), included household-level fixed-effects estimators, and clustered standard errors at the household to consider within-household error correlation.

To identify where and to what extent intake inequities are present, we compared men’s and women’s probabilities of consuming a moderate or high level of healthy food groups using linear probability models (LPM). The LPMs included an interaction effect between food group and gender and included the same adjustments as the OLS models.

### Sensitivity and robustness tests

We applied the following sensitivity and robustness checks:

1. Tested CAR sensitivity to: women’s PAL categorization to evaluate the extent findings would differ in the case of underestimated PALs for women; minimum and maximum additional caloric requirements (+0–400 kcal) for women breastfeeding children aged 12 mo or older; and, for the farm-household sample (body weights were collected only in ANGeL), alternative EER calculations (EER calculated from mean population weights versus individual body weights using the FAO factorial method [19]);
2. Assessed whether gender patterns differ by pregnancy and lactation status (men compared with NPNL women, pregnant women, and lactating women), informed in the studies by Harris-Fry et al. [25] and Wable Grandner et al. [26];
3. Evaluated whether findings were consistent at other survey points using control group 24HR data from the TMRI and ANGeL trials (available from 2012, 2013, and 2014 for TMRI and 2016 and 2018 from ANGeL);
4. Checked for potential recall gaps by comparing female-reported 24HR with male-reported 7DDR of food consumed outside the home;
5. Assessed whether our results were robust to alternative estimation models [models with household fixed effects (as described earlier) compared with models with covariates of household characteristics and village fixed effects].

These sensitivity and robustness tests were decided on a posteriori and can be found in **Section 2** of the **Appendix** (Tables A1–A11).

### Supplemental analyses

In the supplemental analysis found in the Appendix, **Section 3** (Table A12 and Figures A7–A12), we included all individuals older than 2 y and compared dietary indicators between adults (19–49 y), family elders [parents and parents-in-law of the household head (19 y or older] and adults older than 49 y), adolescents (aged 10–18 y), and children (aged 2–9 y). We used OLS regressions to compare dietary indicators across household member types, with adjustments for the same specifications as earlier models. Interaction effects between gender and household member type were used to test whether the effect of gender depended on one’s household position. The dietary indicators we compared included caloric intake, CAR, and DDS. GDQS and probabilities of moderate or high consumption were not included in this supplemental analysis because they have not been validated for individuals younger than 15 y.

We also conducted supplemental tests of men’s and women’s probabilities of unhealthy food group consumption (**Section 4** of the Appendix; Figures A13 and A14).

### Ethical approval and consent

The parent studies, TMRI and ANGeL, received ethical approval from the institutional review board of the International Food Policy Research Institute, Washington DC. The Bangladesh Ministry of Food and Disaster Management and the Ministry of Agriculture, Government of Bangladesh, reviewed the TMRI and ANGeL studies, respectively, and issued Letters of Authorization to conduct the surveys. Consent to participate in the study was received orally from respondents, and this consent was witnessed and formally recorded. The TMRI study was registered with clinicaltrials.gov (study ID: NCT02237144) and the ANGeL study on the Registry for International Development Evaluations (RIDIE-STUDY-ID-5afbe43292b4c).

## Results

### Characteristics of the study samples

Table 1 describes the households and individuals in the samples of ultrapoor households and farm households. In the ultrapoor sample, approximately one-fifth of households owned land (20%) and approximately half of the households were food insecure (46% had an FCS of <42) [27]. The mean per capita monthly household expenditure was 1362 taka (approximately United States $16 in May 2022). Approximately half of the respondents did not have formal schooling (56% of men and 48% of women) and less than half were literate (39% of men and 47% of women). Most adults reported physically demanding occupations (with 62% of men and 53% of women classified as experiencing a high level of physical activity) with the most common occupations being agriculture day labor for men (25%) and homemaking for women (35%) (see Table A17 for descriptives on PAL by gender and occupation). Few women in the ultrapoor sample were pregnant (1%), but most women were lactating at the time of the study (68%). Differences in characteristics by gender were statistically significant.

**TABLE 1:** Characteristics of households and individuals in ultrapoor households and farm households.

	Ultrapoor households			Farm households		
Household characteristics	*N* = 4992			*N* = 4000		
No. of household members	5.17 ± 1.58			5.46 ± 2.07		
Female-headed household	481 (9.6)			158 (4.0)		
Household owns land	1023 (20.5)			3645 (91.1)		
Per capita monthly household expenditure (taka)	1362.38 ± 513.09			3254.24 ± 1486.93		
FCS	47.31 ± 16.28			68.68 ± 17.03		
Low FCS (FCS < 42)^1^	2276 (45.6)			208 (5.2)		
Individual characteristics^2^	Men	Women	Total	Men	Women	Total
	*N* = 5781	*N* = 7189	*N* = 12,970	*N* = 6016	*N* = 6838	*N* = 12,854
Age (y)	37.41 ± 14.97	33.04 ± 14.74	34.99 ± 15.00	37.85 ± 16.13	34.22 ± 16.27	35.92 ± 16.30
Highest level of education achieved						
No schooling	3234 (56.0)	3477 (48.4)	6711 (51.7)	1616 (26.9)	1818 (26.6)	3434 (26.7)
Completed preschool	67 (1.2)	71 (1.0)	138 (1.1)	34 (0.6)	33 (0.5)	67 (0.5)
Completed some or all primary school	1545 (26.7)	2058 (28.6)	3603 (27.8)	1874 (31.2)	1751 (25.6)	3625 (28.2)
Completed some or all secondary or postsecondary school	934 (16.2)	1583 (22.0)	2517 (19.4)	2492 (41.4)	3236 (47.3)	5728 (44.6)
PAL						
Light	1788 (30.9)	3267 (45.4)	5055 (39.0)	2057 (34.2)	4859 (71.1)	6916 (53.8)
Moderate	401 (6.9)	89 (1.2)	490 (3.8)	109 (1.8)	11 (0.2)	120 (0.9)
Heavy	3592 (62.1)	3833 (53.3)	7425 (57.2)	3850 (64.0)	1968 (28.8)	5818 (45.3)
Literate (can read and sign name)	2251 (38.9)	3378 (47.0)	5629 (43.4)	4110 (68.3)	4762 (69.6)	8872 (69.0)
Age group						
Adolescent (15–18 y)	320 (5.5)	380 (5.3)	700 (5.4)	404 (6.7)	537 (7.9)	941 (7.3)
Adult (19+ y)	5461 (94.5)	6809 (94.7)	12,270 (94.6)	5612 (93.3)	6301 (92.1)	11,913 (92.7)
Relation to the household head						
Household head	4215 (72.9)	477 (6.6)	4692 (36.2)	3826 (63.6)	155 (2.3)	3981 (31.0)
Husband/wife	15 (0.3)	4462 (62.1)	4477 (34.5)	5 (0.1)	3762 (55.0)	3767 (29.3)
Son/daughter	1025 (17.7)	430 (6.0)	1455 (11.2)	1359 (22.6)	394 (5.8)	1753 (13.6)
Daughter-in-law/son-in-law	27 (0.5)	839 (11.7)	866 (6.7)	22 (0.4)	1193 (17.4)	1215 (9.5)
Father/mother	313 (5.4)	725 (10.1)	1038 (8.0)	373 (6.2)	874 (12.8)	1247 (9.7)
Father-in-law/mother-in-law	27 (0.5)	96 (1.3)	123 (0.9)	10 (0.2)	51 (0.7)	61 (0.5)
Brother/sister	131 (2.3)	59 (0.8)	190 (1.5)	325 (5.4)	129 (1.9)	454 (3.5)
Brother-in-law/sister-in-law	131 (2.3)	59 (0.8)	190 (1.5)	10 (0.2)	179 (2.6)	189 (1.5)
Pregnant		81 (1.1)			104 (1.5)	
Lactating		4866 (67.7)			3940 (57.6)	
BMI^3^ (kg/m^2^)				20.6 ± 3.1	21.1 ± 3.6	20.9 ± 3.4
Most common occupations	Agricultural day labor (25.2%)	Homemaker (34.7%)		Farmer on own land (31.4%)	Homemaker (60.2%)	
	Rickshaw/van pulling (8.2%)	Raising poultry (28.4%)		Sharecropper/tenant (16.5%)	Raising poultry (16.9%)	
	Sharecropper/tenant (8%)	Raising livestock (21.4%)		Agricultural day labor (7.2%)	Student (4.2%)	

Values presented are mean ± SD or *n* (%).

1 The Bangladesh-specific threshold for an “acceptable” level of food consumption is 42 of 112 [27].

2 Age, education level, and PAL were significantly different (*P* < 0.01) between men and women in both samples; literacy status was significantly different only in the ultrapoor household sample; BMI was significantly different in the farm-household sample (male anthropometric measurements were not taken in the ultrapoor household sample) (Transfer Modality Research Initiative study).

3 Anthropometric information was available only from 1619 men and 1859 women in the ANGeL sample.

Different from the ultrapoor sample, most farm households owned land (91%) and few were food insecure (5% had an FCS of <42), and the mean per capita monthly household was 3254 taka (approximately United States $38.5 in May 2022). Approximately, a quarter of the farm-household respondents did not have formal schooling (27% of men and women), and most were literate (68% of men and 70% of women). Many adults in the farm households reported physically demanding occupations (64% of men and 29% of women are classified as experiencing a high level of physical activity). Farming was the most common occupation for men (31% of men reported working on their own farm, and 17% were sharecroppers), and homemaking was the most common for women (60% of women). Few women in this sample were pregnant (<2%), but most were lactating (58%). The mean BMI (in kg/m^2^) was 20.6 for men and 21.1 for women (excluding pregnant women). All individual characteristics except literacy prevalence were significantly different between men and women.

### Analysis of men’s and women’s diets

Women’s mean unadjusted daily caloric intake was significantly lower than that of men in both the ultrapoor households and farm households (men from the ultrapoor households consumed 2715 kcal, whereas women consumed 2303 kcal; men from the farm households consumed 2497 kcal, whereas women consumed 2220 kcal) (Table 2). When we considered caloric requirements based on PAL, sex, age, pregnancy, and lactation status, men in the ultrapoor sample consumed 94% of caloric needs and women 92% (equivalent to CAR values of 0.94 and 0.92, respectively). In the farm households, men consumed 87% of caloric needs, whereas women consumed 97% of needs. All gender differences were significant at *P* < 0.01.

**TABLE 2:** Unadjusted mean values of dietary indicators.

	Ultrapoor households			Farm households		
	Men	Women	Men–women difference	Men	Women	Men–women difference
Caloric intake (kcal)	2714.54	2303.37	411.17∗∗∗	2497.34	2220.25	277.09∗∗∗
CAR	0.94	0.92	0.016∗∗∗	0.87	0.97	−0.10∗∗∗
DDS	3.92	3.75	0.17∗∗∗	4.56	4.46	0.10∗∗∗
GDQS	5.99	5.51	0.48∗∗∗	8.39	8.07	0.32∗∗∗
Mean probability of moderate or high level of healthy GDQS food group consumption (%)	18.49	17.67	0.82∗∗∗	25.27	24.78	0.49∗∗∗

Abbreviations: CAR, caloric adequacy ratio; DDS, dietary diversity score; GDQS, global diet quality score.

∗∗∗*P* < 0.01.

Diet quality scores (unadjusted) were lower in the ultrapoor sample than those in the farm-household sample and lower for women than for men. As seen in Table 2, men in the ultrapoor households consumed 3.92 food groups (of the 10) and recorded a GDQS score of 5.99 (of the 49), whereas women consumed 3.75 food groups and recorded a GDQS score of 5.51. In the sample of farm households, men consumed 4.56 food groups and reported a GDQS score of 8.39, whereas women consumed 4.46 food groups and reported a GDQS score of 8.07. When we compared the probability of consuming moderate or high levels of healthy food groups, we found that men in the ultrapoor sample showed 18.5% probability and women 17.7% probability, and men and women in farm households showed probabilities of 25.3% and 24.8%, respectively. All gender differences were statistically significant at *P* < 0.01. In both samples, we rejected the null that dietary quality outcomes were equal between men and women.

Figures A1–A6 (Appendix, **Section 1**) display the distributions of unadjusted dietary indicator scores for men and women. In both samples, <50% of the men and women scored within an adequate range for any indicator. For CAR scores, >60% of individuals in the ultrapoor and farm-household samples consumed less than their EER. Approximately 70% of men and women in the ultrapoor sample and 50% of the farm-household sample consumed <5 food groups, the DDS threshold value for a minimally diverse diet. Similarly, 95% or more of individuals in both samples scored <15 on the GDQS, the cutoff value for high risk of nutrient inadequacy and chronic disease.

Next, we tested for gender differences in dietary indicators with household-level fixed effects and other adjustments as described earlier. As seen in Table 3, gender differences slightly changed in magnitude and significance compared with unadjusted results, but differences remained in the same direction. In both samples, women consumed significantly fewer calories than men (ultrapoor households: women consumed 441 kcal less; farm households: women consumed 298 kcal less). Relative to their caloric requirements, women in ultrapoor households consumed less than men (women’s CAR was 0.017 or 1.7% lower, a significant difference), whereas women in farm households consumed more than men (women’s CAR was 10% higher, a significant difference). Women scored lower on all measures of dietary quality (ultrapoor households: women showed a lower DDS by 0.16 food groups, scored 0.55 lower for GDQS, and recorded 0.76% lower probability of consuming moderate or high levels of healthy food groups; farm households: women showed a lower DDS by 0.099 food groups, scored 0.34 lower for GDQS, and recorded 0.51% lower probability of consuming moderate or high levels of healthy food groups). All gender differences were significant at *P* < 0.01.

**TABLE 3:** Gender differences in dietary indicators: OLS estimates

Variable	Ultrapoor households					Farm households				
	Caloric intake	CAR	DDS	GDQS	Probability of adequate consumption (%)	Caloric Intake	CAR	DDS	GDQS	Probability of adequate consumption (%)
Women	−441.13∗∗∗	−0.017∗∗∗	−0.16∗∗∗	−0.55∗∗∗	−0.76∗∗∗	−297.89∗∗∗	0.10∗∗∗	−0.099∗∗∗	−0.34∗∗∗	−0.51∗∗∗
Age at baseline (ln)	−148.16∗∗∗	0.053∗∗∗	0.19∗∗∗	−0.084∗∗	−0.28∗∗∗	−200.11∗∗∗	0.010	0.092∗∗∗	−0.17∗∗∗	−0.35∗∗∗
Constant	3245.80∗∗∗	0.75∗∗∗	3.27∗∗∗	6.32∗∗∗	19.44∗∗∗	3206.43∗∗∗	0.83∗∗∗	4.24∗∗∗	8.99∗∗∗	26.50∗∗∗
No. of observations	12,970	12,970	12,970	12,970	12,970	12,854	12,854	12,854	12,854	12,854
Adjusted *R*^2^	0.26	0.017	0.053	0.071	0.026	0.15	0.075	0.018	0.025	0.011

Models include household fixed effects and household-clustered standard errors.

Abbreviations: CAR, caloric adequacy ratio; DDS, dietary diversity score; GDQS, global diet quality score.

∗∗*P* < 0.05; ∗∗∗*P* < 0.01.

When we disaggregated by healthy food groups, we found that the probabilities of consuming moderate or high levels of healthy food groups were nearly equal between men and women in both samples for most food groups, as seen with the overlapping confidence intervals (TABLE 4, TABLE 5). The few exceptions were in ultrapoor sample, where women showed 1%–4% lower probabilities of consuming moderate to high levels of fish and shellfish, other vegetables, whole grains, liquid oils, and high-fat dairy. In both samples, few respondents consumed moderate or high levels of nutrient-dense food items such as animal-sourced foods (except fish in farm households), legumes, fruits, or dark leafy green vegetables.

**TABLE 4:** Probability of medium or high consumption by healthy GDQS food group and gender in ultrapoor households: LPM estimates

Food group	Men			Women		
	Predicted probability of medium or high consumption (%)	95% CI		Predicted probability of medium or high consumption (%)	95% CI	
Citrus fruits	0.05	<0.01	0.28	0.16	<0.01	0.36
Deep orange fruits	0.81	0.48	1.14	1.03	0.71	1.35
Other fruits	5.71	5.01	6.41	5.75	5.09	6.41
Dark green leafy vegetables	25.70	24.32	27.08	28.35	27.02	29.69
Cruciferous vegetables	3.02	2.40	3.65	2.46	1.98	2.95
Deep orange vegetables	10.34	9.34	11.34	10.30	9.38	11.21
Other vegetables	81.14	79.95	82.33	77.16	75.97	78.35
Legumes	27.97	26.55	29.39	25.86	24.55	27.17
Deep orange tubers	<0.01	<0.01	0.16	0.04	<0.01	0.24
Nuts and seeds	0.14	<0.01	0.38	0.32	0.08	0.56
Whole grains	17.19	15.99	18.39	14.19	13.15	15.23
Liquid oils	97.18	96.70	97.65	96.33	95.84	96.82
Fish and shellfish	48.55	47.03	50.08	44.97	43.57	46.38
Poultry and game meat	3.35	2.75	3.96	3.01	2.49	3.53
Low-fat dairy	<0.01	<0.01	0.20	0.07	<0.01	0.27
Eggs	0.10	<0.01	0.35	0.14	<0.01	0.35
High-fat dairy	8.13	7.28	8.98	6.48	5.80	7.17
Red meat	2.54	2.02	3.06	2.20	1.76	2.64

This model contained an interaction effect between food group and gender and adjusted for age (years, log transformed) and household fixed effects. Standard errors are clustered at the household level. Predicted probabilities and CIs are displayed for ease of interpretation. Full interaction model results are available on request.

Abbreviations: GDQS, global diet quality score.

**TABLE 5:** Probability of medium or high consumption by healthy GDQS food group and gender in farming households: LPM estimates

Food group	Men			Women		
	Predicted probability of medium or high consumption (%)	95% CI		Predicted probability of medium. or high consumption (%)	95% CI	
Citrus fruits	1.57	1.10	2.04	1.81	1.35	2.27
Deep orange fruits	0.17	<0.01	0.44	0.33	0.04	0.62
Other fruits	6.76	5.91	7.60	7.04	6.24	7.84
Dark green leafy vegetables	35.18	33.57	36.79	34.97	33.43	36.52
Cruciferous vegetables	36.78	35.12	38.43	36.03	34.44	37.62
Deep orange vegetables	2.30	1.73	2.87	2.20	1.66	2.74
Other vegetables	94.62	93.90	95.34	93.70	92.96	94.45
Legumes	26.67	25.17	28.17	25.61	24.16	27.07
Deep orange tubers	0.01	<0.01	0.25	0.05	<0.01	0.29
Nuts and seeds	0.61	0.26	0.96	0.70	0.35	1.04
Whole grains	27.53	25.95	29.12	25.42	23.94	26.91
Liquid oils	99.39	99.09	99.69	99.20	98.89	99.52
Fish and shellfish	76.39	75.00	77.77	75.78	74.42	77.14
Poultry and game meat	11.84	10.71	12.97	11.75	10.64	12.86
Low-fat dairy	0.02	<0.01	0.27	0.02	<0.01	0.25
Eggs	16.65	15.44	17.85	15.28	14.13	16.42
High-fat dairy	12.66	11.58	13.74	11.30	10.33	12.27
Red meat	5.49	4.70	6.29	4.92	4.18	5.66

This model contained an interaction effect between food group and gender and adjusted for age (years, log transformed) and household fixed effects. Standard errors are clustered at the household level. Predicted probabilities and confidence intervals (CIs) are displayed for ease of interpretation. Full interaction model results are available on request.

Abbreviations: CAR, caloric adequacy ratio; DDS, dietary diversity score; GDQS, global diet quality score.

### Sensitivity and robustness tests

Our CAR results were moderately sensitive to how we classified women’s PAL, estimated breastfeeding requirements, and whether we calculated EER with individual or population-mean body weights. However, gender differences in CARs remained in the same direction and similar in magnitude (maximum of a 5% CAR male advantage in the most extreme scenario where no women had a low PAL). The one exception was the lactation requirements sensitivity test in the sample of ultrapoor households, where when we omitted the additional 400-kcal energy requirement for women breastfeeding infants aged 12 mo or older, women’s CAR became higher than men’s (by 7.2%, a significant difference) (Tables A1–A3 for CAR sensitivity tests).

Results were also sensitive to pregnancy and lactation status, with patterns differing slightly between samples. In the ultrapoor sample, NPNL women showed higher CARs than men, whereas all other dietary indicators remained lower than men regardless of pregnancy or lactation status. In the farm households, lactating women scored higher than men on all diet indicators (CAR, DDS, GDQS, and probability of medium or high consumption of healthy foods), except caloric intake. NPNL and pregnant women in the farm-household sample showed higher CARs but lower caloric intakes and diet quality scores than men (similar to earlier results) (Table A4 for full results).

The main results were consistent across survey points (Tables A6–A9). Among the control groups, women showed lower diet quality scores but similar or higher CARs than men across surveys. Over time, however, gender differences in diet quality scores became smaller while women’s CAR grew increasingly higher than men’s (including in the ultrapoor sample). Across all surveys and both samples, men and women from the control groups showed nondifferent probabilities of consuming moderate or high levels of all healthy foods. The one exception was in the farm households at endline, where women showed 4.3% higher probability of consuming a moderate or high level of other fruits.

When we compared the reported household consumption of food outside the home between the female-reported 24HR survey and the male-reported 7DDR, we found a mean difference of 16.9 g/d/household between surveys. The 7DDR reported slightly higher household intakes of certain deep-fried foods, refined grains, and sweets (Table A11).

Results were also robust to alternative estimations models. Models including covariates for household characteristics and village fixed effects instead of household fixed effects produced similar results (Table A10).

### Supplemental tests

The observed gender patterns were similar across household member types: males recorded higher caloric intake than females; females recorded higher or equal CARs (except female adults in ultrapoor households who showed lower CARs) and males consumed more food groups than females. Gender differences were the smallest among children. Adult men consumed the most calories and number of food groups, whereas adolescent and elder women in ultrapoor households and adult and elder women in farm households had the highest CARs. The full results of dietary indicator scores by household member type are available in Table A12 and Figures A7–A12.

When we compared the consumption of unhealthy foods between men and women, we found that men had higher probabilities of consuming moderate or high amounts of sugar-sweetened beverages and sweets and ice cream. Apart from white roots and tubers and refined grains and baked goods, consumption of unhealthy foods was low across samples (Figures A13 and A14).

## Discussion

We revisit the issue of intrahousehold food allocation in South Asia, drawing on 2 data sources to assess whether rural Bangladeshi women are discriminated against. We find that, as have earlier studies, a promale allocation bias in energy intake exists in both the ultrapoor households and farm households. However, when we consider physiological sex differences and PALs, the male advantage in caloric consumption disappeared. In reference to their caloric needs, women in ultrapoor households consumed women nearly equal (<2% different than men) compared with men, whereas women in farm households consumed more.

We compared diet quality between men and women to determine whether within-household discrimination manifested itself in other elements of diets, particularly the consumption of higher-quality foods. Although the gender differences in dietary quality scores were statistically significant, the magnitudes of these differences were negligible in both samples: women scored <1% lower than men in the aggregate diet quality indicators (DDS, GDQS, and probability of consuming moderate or high levels of healthy foods) and men and women showed similar likelihoods of consuming sizable portions of most healthy food groups. Notably, however, men and women alike showed poor overall diet quality and low consumption of all healthy GDQS food groups except other vegetables and fish.

Our supplemental analysis showed that although the CAR values were sensitive to how energy requirements were estimated, the pattern of women consuming nearly equal or more in reference to their needs compared with men remained unchanged. The only exception was pregnant women and lactating women in the sample of ultrapoor households who showed more calorically deficient diets than men. However, this is likely not explained by gender bias in intrahousehold food allocation. If that were the case, we would expect all women to have appreciably poorer diets than men. However, NPNL women in both samples and pregnant and lactating women from farm households showed higher caloric adequacy than men. In addition, lactating women in farm households recorded higher diet quality than men. Instead, the poorer diets of pregnant and lactating women in ultrapoor households may be due to their inability to increase intake to meet the added nutrition demands of pregnancy or lactation owing to food insecurity. Undernourishment of pregnant and lactating women may be further exacerbated by food aversion, loss of appetite, illness, or cultural beliefs related to dietary restriction during pregnancy and postpartum, although recent evidence suggests a generational shift in this belief [26,[28], [29], [30], [31]].

When we included children in our analysis and compared diets between household member types, we found that the gender patterns were consistent in both samples (females showed higher caloric adequacy of their diets but slightly lower dietary diversity), but gender differences were generally most pronounced among adults and elders. An exception to this pattern was adult women in the ultrapoor sample who showed slightly lower caloric adequacy than adult men, which was likely driven by the high representation of lactating women (68%) in the sample who presented with lower CARs, as discussed earlier.

Although the samples included in our study are not entirely representative of Bangladesh, rather an oversampling of households at different points of the income and food-security distribution in rural Bangladesh, our findings are in line with the study by Ahmed et al., who used representative data from the 2011 and 2018 Bangladesh Integrated Household Survey to study food consumption trends in rural Bangladesh [15]. Their analysis showed that, in 2011, women experienced slightly lower caloric adequacy and dietary quality than men, but by 2018, women had narrowly surpassed men in multiple dietary indicators. This trend is similar to what we observed in our control group analysis over the successive survey time points: the minor male advantage in diet quality diminished over time.

Compared with rural Bangladeshi population averages in the study by Ahmed et al., the ultrapoor households in our sample had poorer quality of diets (lower DDS), whereas the farm households had better diet quality, as expected. However, our estimates of caloric insufficiency for both men and women were higher in both of our samples (in the study by Ahmed et al., 14%–34% of adolescent and adult men and 13%–42% of adult and adolescent women were considered to have insufficient energy intake). This is likely due to our study’s higher cutoff for caloric insufficiency, higher adjustments for PAL, and potential differences in occupations (and, therefore, differences in energy requirements) compared with the general rural Bangladeshi population. In that sense, our study estimates the upper bounds of caloric inadequacy in rural Bangladesh. Regardless of these differences, both studies suggest that diets are generally suboptimal but equitable between genders in rural Bangladesh.

This study has several strengths. Our analysis included a combination of measures that allowed us to assess and compare diets on multiple dimensions. We not only tested whether diets are different in quality and quantity but also examined whether these differences are meaningful in magnitude by comparing intakes with caloric requirements and portion sizes. The patterns we observed were similar across the 2 study populations, representing 4 different regions and multiple time points within the past decade in Bangladesh. Our results also remained robust to multiple sensitivity tests and alternative caloric requirements calculations and estimation models, giving further confidence to our results.

Our study also has limitations. First, we analyzed diets using a single-day 24-h recall, which is prone to random error from day-to-day variation in food consumption. To determine whether our gender comparisons were affected by this, we repeated our analysis with the control groups from both samples at other survey points. Our findings were similar across all 5 control group samples: women consumed near equal or more relative to their caloric needs than men, whereas men showed higher diet quality scores than women, the differences were minimal (<1% differences) and did not reflect appreciable consumption disparities of nutrient-rich foods.

Second, the 24HR dietary intake information for each household member was provided by women who were responsible for preparing and serving food. Because men in this setting typically have higher resource control and movement outside the house, it is possible that men may have purchased and consumed food outside the home without their spouse’s knowledge, leading women to underreport male dietary intake. To identify whether this potential bias was present in our study, we used TMRI survey data to compare the reported household consumption of food outside the home between the 24HR survey and the 7DDR, which was reported by the male household head. In doing so, we found a small mean difference of 16.9 g/d per household between the surveys. The 7DDR reported slightly higher household intakes of certain deep-fried foods, refined grains, and sweets. Because the differences between surveys of reported food consumed outside the home were small and these food items were from unhealthy food groups, we do not expect this potential reporting bias to affect the study’s findings.

Third, because anthropometric measures were not collected for men in the TMRI study (sample of ultrapoor households), we estimated adult caloric requirements using population body weights for Bangladeshi adults to avoid precision imbalances between men and women and between the 2 study samples. However, using population weights instead of individual weights limits the precision of our CAR estimates. To assess how this limitation affected the CAR results, we compared CAR estimations based on population weights compared with individual weights in the farm-household sample (ANGeL study), which had weight data from both men and women. Although including individual weights increased the CAR values for men and women and decreased the gender difference—indicating that our population weight-based caloric requirements overestimated requirements—women still consumed more in reference to their caloric needs than men. Thus, although the inclusion of individuals’ weights increased the precision of CAR values, it did not change our understanding of intrahousehold food allocation patterns.

Finally, dietary indicators and recommendations for pregnant and lactating women are limited. The DDS and GDQS have not been validated for micronutrient adequacy in pregnant or lactating women. Therefore, it is possible that the gender differences we found with our diet quality indicators do not reflect differences in nutrient adequacy for women in these life stages. We do not have information on supplement intake or multiple days of 24HR recalls to assess the micronutrient adequacy of men’s and women’s diets in our samples.

Moreover, to our knowledge, there are no energy intake recommendations for women breastfeeding after 12 mo. Our CAR calculations used the same recommended caloric intake for women breastfeeding children aged 6–12 mo and women breastfeeding children aged 12–14 mo (400 additional calories). However, the required intake of women breastfeeding a child aged 12–14 mo is likely less because the volume of milk production typically declines in later months of breastfeeding as the child weans from breastfeeding to solid foods [19]. Accordingly, we may have overestimated caloric requirements for lactating women and, therefore, underestimated CAR differences between men and women, particularly among the ultrapoor households where 68% of women were lactating and among lactating women where 56% were breastfeeding children aged 12–24 mo.

In these samples of ultrapoor and farm households rural Bangladeshi, the diets of adult men and women were relatively equitable but suboptimal. Although the study results do not provide evidence of male favoritism in household food allocation, it highlights that poor diet quality—driven mostly by high consumption of refined grains and low consumption of healthy food groups—is a pressing concern for men and women alike across socioeconomic stratum of rural Bangladesh. These findings provide both a hope and a challenge for policy and programmatic action against malnutrition in rural Bangladesh.

## Funding

This research was supported by the National Institutes of Health (award T32-DK007158). TMRI received funding from the German Ministry for Economic Cooperation and Development; UK Department for International Development (DFID); Swiss Agency for Development and Cooperation (SDC); United Nations Development Programme (UNDP); the World Food Programme (WFP); and the United States Agency for International Development (USAID). The ANGeL study was supported by the USAID (Grant Number EEM-G-00-04-00013-00); the Gender, Agriculture, and Assets Project–Phase 2 (GAAP2) supported by the Bill & Melinda Gates Foundation; and the CGIAR Research Program on Agriculture for Nutrition and Health (A4NH). The supporting sources had no involvement or restrictions regarding publication.

## Author disclosures

The authors report no conflicts of interest.

## Data availability

Individual participant data that underlie the results reported in this article collected during the trial will be available immediately after publication with no end date after deidentification. Data will be available to those who wish to access the data and study protocols, statistical analysis plan, and analytic code will be shared.

## Appendix A Supplementary data

Multimedia component1 Multimedia component1
