# Supplementary material for: Diets of Men and Women in Rural Bangladesh Are Equitable but Suboptimal
Source: Curr Dev Nutr. 2023 Jun 2;7(7):100107. doi: 10.1016/j.cdnut.2023.100107 (PMC10310464; doi:10.1016/j.cdnut.2023.100107)
Supplement: Multimedia component1 [file mmc1.docx]

**Appendix**

[1 Distributions of Dietary Scores 3](#_Toc136041729)

[Figure A1: Distribution of CAR Scores, Ultra-Poor Households 3](#_Toc136041730)

[Figure A2: Distribution of CAR Scores, Farm Households 4](#_Toc136041731)

[Figure A3: Distribution of DDS Scores, Ultra-Poor Households 5](#_Toc136041732)

[Figure A4: Distribution of DDS Scores, Farm Households 6](#_Toc136041733)

[Figure A5: Distribution of GDQS Scores, Ultra-Poor Households 7](#_Toc136041734)

[Figure A6: Distribution of GDQS Scores, Farm Households 8](#_Toc136041735)

[2. Sensitivity Tests 9](#_Toc136041736)

[Table A1: CAR Sensitivity to Women’s PAL Categorization, OLS Estimates 9](#_Toc136041737)

[Table A2: CAR Sensitivity to Estimated Lactation Requirements, OLS estimates 9](#_Toc136041738)

[Table A3: CAR Sensitivity to Population-Based Weight vs. Individual Weight (Farm Households) 10](#_Toc136041739)

[Table A4: Gender Differences in Dietary Indicators by Pregnancy and Lactation Status 11](#_Toc136041740)

[Table A5: Months of Lactation 11](#_Toc136041741)

[Table A6: Control Group Gender Differences in Dietary Indicators, at Baseline, Midline, and Endline from Ultra-Poor Household Sample (TMRI) 12](#_Toc136041742)

[Table A7: Control Group Gender Differences in Dietary Indicators, at Baseline and Endline from Farm Household Sample (ANGeL) 13](#_Toc136041743)

[Table A8: Control Group Probabilities of Medium or High Consumption by Healthy GDQS Food Group and Gender at Baseline, Midline, and Endline from Ultra-Poor Household Sample (TMRI) 14](#_Toc136041744)

[Table A9: Control Group Probabilities of Medium or High Consumption by Healthy GDQS Food Group and Gender at Baseline and Endline from Farm Household Sample (ANGeL) 16](#_Toc136041745)

[Table A10: Gender Differences in Dietary Indicators, Alternative Estimation Model (Household Covariates, Village FE, and Village clustered SE) 18](#_Toc136041746)

[Table A11: Reported Consumption of Food Outside the Home, 24HR versus 7DDR Surveys (Ultra-Poor Households) 20](#_Toc136041747)

[3. Dietary Indicator Scores, by Household Member Type 21](#_Toc136041748)

[Table A12: Differences Dietary Indicators in Dietary by Household Member Type 21](#_Toc136041749)

[Figure A7: Caloric Intake by Household Member Type in Ultra-Poor Households, OLS Predicted Values 22](#_Toc136041750)

[Figure A8: Caloric Intake by Household Member Type in Farm Households, OLS Predicted Values 23](#_Toc136041751)

[Figure A9: CAR by Household Member Type in Ultra-Poor Households, OLS Predicted Values 24](#_Toc136041752)

[Figure A10: CAR by Household Member Type in Farm Households, OLS Predicted Values 25](#_Toc136041753)

[Figure A11: DDS by Household Member Type in Ultra-Poor Households, OLS Predicted Values 26](#_Toc136041754)

[Figure A12: DDS by Household Member Type in Farm Households, OLS Predicted Values 27](#_Toc136041755)

[4. Additional Tests 28](#_Toc136041756)

[Figure A13: Probability of Medium or High Consumption of Unhealthy Food Groups in Ultra-Poor Households, LPM Predicted Values 28](#_Toc136041757)

[Figure A14: Probability of Medium or High Consumption of Unhealthy Food Groups in Farm Households, LPM Predicted Values 29](#_Toc136041758)

[5. Further Information on Dietary Measurement Methods 30](#_Toc136041759)

[Table A13: Estimated Energy Requirements for Adolescents and Adults based on Physical Activity Level 30](#_Toc136041760)

[Table A14: Adjustments in Energy Requirements for Pregnancy and Lactation 30](#_Toc136041761)

[Table A15: Estimated Energy Requirements for Children and Young Adolescents 31](#_Toc136041762)

[Table A16: GDQS Food Groups and Scoring 32](#_Toc136041763)

[Table A17: PAL by Occupation and Gender 33](#_Toc136041764)

[6. References 36](#_Toc136041765)

# Distributions of Dietary Scores

### Figure A1: Distribution of CAR Scores, Ultra-Poor Households

### Figure A2: Distribution of CAR Scores, Farm Households

### Figure A3: Distribution of DDS Scores, Ultra-Poor Households

### Figure A4: Distribution of DDS Scores, Farm Households

### Figure A5: Distribution of GDQS Scores, Ultra-Poor Households

### Figure A6: Distribution of GDQS Scores, Farm Households

# Sensitivity Tests

### Table A1: CAR Sensitivity to Women’s PAL Categorization, OLS Estimates

|  | **Ultra-Poor Households** | | | **Farm Households** | | |
| --- | --- | --- | --- | --- | --- | --- |
| **Variable** | CAR with guideline-based PAL for occupations | CAR with female home keepers in at least moderate PAL category | CAR with all women in at least moderate PAL category | CAR with guideline-based PAL for occupations | CAR with female home keepers in at least moderate PAL category | CAR with all women in at least moderate PAL category |
|  |  |  |  |  |  |  |
| Women | -0.017*** | -0.041*** | -0.054*** | 0.10*** | 0.051*** | 0.038*** |
| Age at Baseline (ln) | 0.053*** | 0.032*** | 0.022*** | 0.010 | -0.025*** | -0.029*** |
| Constant | 0.75*** | 0.83*** | 0.86*** | 0.83*** | 0.95*** | 0.97*** |
| Number of Observations | 12970 | 12970 | 12970 | 12854 | 12854 | 12854 |
| Adj. R-squared | 0.017 | 0.023 | 0.033 | 0.075 | 0.029 | 0.021 |

Notes: * p <0.10; **p <0.05; ***p<0.01. Models include household fixed effects and household-clustered standard errors. All men and women are included in the above analysis.

### Table A2: CAR Sensitivity to Estimated Lactation Requirements, OLS estimates

|  | **Ultra-Poor Households** | | **Farm Households** | |
| --- | --- | --- | --- | --- |
| **Variable** | CAR where caloric intake has a +400kcal adjustment for women who reported breastfeeding children 12+ months | CAR with no adjustments for women breastfeeding children 12+ months | CAR where caloric intake has a +400kcal adjustment for women who reported breastfeeding children 12+ months | CAR with no adjustments for women breastfeeding children 12+ months |
| Women | -0.017*** | 0.072*** | 0.10*** | 0.12*** |
| Age at Baseline (ln) | 0.053*** | -0.005 | 0.010 | 0.001 |
| Constant | 0.75*** | 0.96*** | 0.83*** | 0.86*** |
| Number of Observations | 12970 | 12970 | 12854 | 12854 |
| Adj. R-squared | 0.017 | 0.039 | 0.075 | 0.092 |

Notes: * p <0.10; **p <0.05; ***p<0.01. Models include household fixed effects and household-clustered standard errors. All men and women are included in the above analysis. All men and women are included in the above analysis, including NPNL and pregnant women.

### Table A3: CAR Sensitivity to Population-Based Weight vs. Individual Weight (Farm Households)

| **Variable** | CAR, Estimated with Average Population Weights | CAR, Estimated with Individual Weights |
| --- | --- | --- |
|  |  |  |
| Women | 0.10*** | 0.097*** |
| Age at Baseline (ln) | 0.010 | 0.001 |
| Constant | 0.83*** | 0.91*** |
| Number of Observations | 12854 | 3507 |
| Adj. R-squared | 0.075 | 0.062 |

Notes: * p <0.10; **p <0.05; ***p<0.01. Models include household fixed effects and household-clustered standard errors. Individuals without weight information and pregnant women are excluded.

## Table A4: Gender Differences in Dietary Indicators by Pregnancy and Lactation Status

|  | **Ultra-Poor Households** | | | | | **Farm Households** | | | | | |
| --- | --- | --- | --- | --- | --- | --- | --- | --- | --- | --- | --- |
| **Variable** | Caloric Intake | CAR | DDS | GDQS | Prob. Of Adequate Consumption (%) | Caloric Intake | CAR | DDS | GDQS | Prob. Of Adequate Consumption |  |
|  |  |  |  |  |  |  |  |  |  |  |  |
| Women | -527.54*** | 0.058*** | -0.22*** | -0.58*** | -0.89*** | -383.93*** | 0.16*** | -0.13*** | -0.40*** | -0.61*** |  |
| Lactating | 136.87*** | -0.11*** | 0.082*** | 0.056 | 0.20* | 158.37*** | -0.095*** | 0.059*** | 0.11** | 0.18* |  |
| Pregnant | -80.60 | -0.21*** | -0.016 | 0.010 | 0.11 | -19.52 | -0.11*** | -0.025 | -0.087 | -0.19 |  |
| Age at Baseline (ln) | -94.43*** | 0.006 | 0.22*** | -0.061 | -0.20* | -136.48*** | -0.029*** | 0.12*** | -0.13*** | -0.28** |  |
| Constant | 3056.22*** | 0.92*** | 3.16*** | 6.24*** | 19.15*** | 2981.86*** | 0.97*** | 4.16*** | 8.84*** | 26.26*** |  |
| Number of Observations | 12970 | 12970 | 12970 | 12970 | 12970 | 12854 | 12854 | 12854 | 12854 | 12854 |  |
| Adj. R-squared | 0.27 | 0.057 | 0.055 | 0.071 | 0.026 | 0.16 | 0.10 | 0.019 | 0.026 | 0.011 |  |

Notes: * p <0.10; **p <0.05; ***p<0.01. Models include household fixed effects and household-clustered standard errors. Individuals without weight information and pregnant women are excluded. “Men” is the reference category.

## Table A5: Months of Lactation

|  | Ultra-Poor Households | | Farm Households | |
| --- | --- | --- | --- | --- |
| Age of Breastfed Child (Months Postpartum) | Freq. | Percent (%) | Freq. | Percent (%) |
|  |  |  |  |  |
| 0-6 months | 893 | 18.35 | 1,043 | 26.42 |
| 6-12 months | 1,271 | 26.12 | 1,024 | 25.94 |
| 12-24 months | 2,702 | 55.53 | 1,881 | 47.64 |
|  |  |  |  |  |
| Total | 4,866 | 67.69 (of women in sample) | 3,948 | 36.59 (of women in sample) |

## Table A6: Control Group Gender Differences in Dietary Indicators, at Baseline, Midline, and Endline from Ultra-Poor Household Sample (TMRI)

|  | Variable | Caloric Intake | CAR | DDS | GDQS | Prob. Of Adequate Consumption |
| --- | --- | --- | --- | --- | --- | --- |
| Baseline (2012) |  |  |  |  |  |  |
|  | Women | -454.60*** | -0.011 | -0.18*** | -0.59*** | -0.76*** |
|  | Age at Baseline (ln) | -185.34*** | 0.045** | 0.15*** | -0.18** | -0.47* |
|  | Constant | 3350.18*** | 0.77*** | 3.39*** | 6.67*** | 20.26*** |
|  | Number of Observations | 2656 | 2656 | 2656 | 2656 | 2656 |
|  | Adj. R-squared | 0.24 | 0.010 | 0.045 | 0.069 | 0.022 |
| Midline (2013) |  |  |  |  |  |  |
|  | Women | -442.83*** | 0.035** | -0.15*** | -0.37*** | -0.50*** |
|  | Age at Baseline (ln) | -172.12*** | 0.031 | 0.16*** | -0.10 | -0.53* |
|  | Constant | 3264.01*** | 0.80*** | 3.72*** | 7.23*** | 22.79*** |
|  | Number of Observations | 2256 | 2256 | 2256 | 2256 | 2256 |
|  | Adj. R-squared | 0.25 | 0.009 | 0.043 | 0.033 | 0.013 |
| Endline (2014) |  |  |  |  |  |  |
|  | Women | -440.13*** | 0.083*** | -0.14*** | -0.27*** | -0.35*** |
|  | Age at Baseline (ln) | -205.86*** | 0.017 | 0.14** | -0.089 | -0.18 |
|  | Constant | 3350.49*** | 0.84*** | 3.60*** | 6.62*** | 20.26*** |
|  | Number of Observations | 2134 | 2134 | 2134 | 2134 | 2134 |
|  | Adj. R-squared | 0.30 | 0.054 | 0.043 | 0.030 | 0.008 |

## Table A7: Control Group Gender Differences in Dietary Indicators, at Baseline and Endline from Farm Household Sample (ANGeL)

|  | Variable | Caloric Intake | CAR | DDS | GDQS | Prob. Of Adequate Consumption |
| --- | --- | --- | --- | --- | --- | --- |
| **Baseline (2016)** |  |  |  |  |  |  |
|  | Women | -280.51*** | 0.09*** | -0.12*** | -0.37*** | -0.54*** |
|  | Age at Baseline (ln) | -173.37*** | 0.018 | 0.10*** | -0.13 | -0.10 |
|  | Constant | 3108.62*** | 0.81*** | 4.26*** | 8.84*** | 25.29*** |
|  | Number of Observations | 2824 | 2824 | 2824 | 2824 | 2824 |
|  | Adj. R-squared | 0.14 | 0.064 | 0.027 | 0.028 | 0.010 |
| **Endline (2018)** |  |  |  |  |  |  |
|  | Women | -356.01*** | 0.18*** | -0.048 | -0.27*** | -0.22 |
|  | Age at Baseline (ln) | -248.22*** | -0.058*** | 0.067 | -0.27*** | -0.53** |
|  | Constant | 3443.50*** | 1.10*** | 4.23*** | 8.96*** | 26.31*** |
|  | Number of Observations | 2261 | 2261 | 2261 | 2261 | 2261 |
|  | Adj. R-squared | 0.24 | 0.26 | 0.006 | 0.020 | 0.006 |

## Table A8: Control Group Probabilities of Medium or High Consumption by Healthy GDQS Food Group and Gender at Baseline, Midline, and Endline from Ultra-Poor Household Sample (TMRI)

|  | **Baseline (2012)** | | | | | |
| --- | --- | --- | --- | --- | --- | --- |
|  | **Men** | | | **Women** | | |
| **Food Group** | Prob. High or Med Cons. (%) | 95% CI | | Prob. High or Med Cons. (%) | 95% CI | |
| Citrus fruits | 0.14 | <0.01 | 0.69 | 0.09 | <0.01 | 0.55 |
| Deep orange fruits | 0.06 | <0.01 | 0.54 | 0.16 | <0.01 | 0.66 |
| Other fruits | 6.51 | 4.80 | 8.22 | 6.24 | 4.68 | 7.81 |
| Dark green leafy vegetables | 24.35 | 21.25 | 27.46 | 26.46 | 23.49 | 29.43 |
| Cruciferous vegetables | 2.77 | 1.55 | 3.99 | 2.05 | 1.13 | 28.88 |
| Deep orange vegetables | 10.76 | 8.48 | 13.04 | 11.18 | 9.06 | 13.30 |
| Other vegetables | 81.87 | 79.31 | 84.44 | 77.78 | 75.21 | 80.34 |
| Legumes | 27.33 | 24.15 | 30.51 | 26.46 | 23.50 | 29.42 |
| Deep orange tubers | <0.01 | <0.01 | 0.43 | 0.02 | <0.01 | 0.46 |
| Nuts and seeds | 0.06 | <0.01 | 0.54 | 0.02 | <0.01 | 0.46 |
| Whole grains | 20.02 | 17.17 | 22.87 | 16.86 | 14.39 | 19.33 |
| Liquid oils | 96.66 | 95.56 | 97.75 | 96.37 | 95.30 | 97.45 |
| Fish and shellfish | 49.76 | 46.36 | 53.16 | 47.22 | 44.06 | 50.37 |
| Poultry and game meat | 3.71 | 2.33 | 5.09 | 3.20 | 2.00 | 4.41 |
| Low-fat dairy | 0.06 | <0.01 | 0.55 | 0.09 | <0.01 | 0.55 |
| Eggs | 0.23 | <0.01 | 0.85 | 0.23 | <0.01 | 0.78 |
| High-fat dairy | 8.13 | 6.21 | 10.04 | 5.77 | 4.32 | 7.22 |
| Red meat | 2.60 | 1.44 | 3.77 | 2.19 | 1.20 | 3.17 |
|  | **Midline (2013)** | | | | | |
|  | **Men** | | | **Women** | | |
| **Food Group** | Prob. High or Med Cons. (%) | 95% CI | | Prob. High or Med Cons. (%) | 95% CI | |
| Citrus fruits | 0.33 | 0.24 | 0.89 | <0.01 | 0.49 | 0.45 |
| Deep orange fruits | 24.46 | 21.44 | 27.47 | 28.23 | 25.40 | 31.06 |
| Other fruits | 5.75 | 4.12 | 7.38 | 4.99 | 3.56 | 6.43 |
| Dark green leafy vegetables | 32.33 | 28.86 | 35.81 | 33.47 | 30.34 | 28.88 |
| Cruciferous vegetables | 2.78 | 1.44 | 4.12 | 2.49 | 1.25 | 3.72 |
| Deep orange vegetables | 11.68 | 9.30 | 14.06 | 11.96 | 9.68 | 14.23 |
| Other vegetables | 82.33 | 79.52 | 85.15 | 78.62 | 75.94 | 81.30 |
| Legumes | 30.80 | 27.31 | 34.28 | 30.27 | 27.12 | 33.41 |
| Deep orange tubers | 0.12 | 0.37 | 0.62 | <0.01 | 0.55 | 0.37 |
| Nuts and seeds | 3.29 | 1.95 | 4.63 | 4.21 | 2.80 | 5.62 |
| Whole grains | 16.59 | 13.80 | 19.37 | 13.21 | 10.94 | 15.48 |
| Liquid oils | 97.36 | 96.08 | 98.64 | 96.93 | 95.85 | 98.02 |
| Fish and shellfish | 47.36 | 43.77 | 50.96 | 45.60 | 42.44 | 48.76 |
| Poultry and game meat | 3.91 | 2.50 | 5.31 | 3.27 | 2.06 | 4.49 |
| Low-fat dairy | 0.12 | 0.37 | 0.62 | <0.01 | 0.55 | 0.37 |
| Eggs | 0.63 | 0.04 | 1.31 | 0.53 | <0.01 | 1.29 |
| High-fat dairy | 12.09 | 9.78 | 14.40 | 11.33 | 9.26 | 13.40 |
| Red meat | 4.52 | 3.08 | 5.96 | 3.90 | 2.59 | 5.21 |
|  | **Endline (2014)** | | | | | |
|  | **Men** | | | **Women** | | |
| **Food Group** | Prob. High or Med Cons. | 95% CI | | Prob. High or Med Cons. | 95% CI | |
| Citrus fruits | 0.05 | <0.01 | 0.57 | 0.29 | <0.01 | 0.78 |
| Deep orange fruits | 0.38 | <0.01 | 1.08 | 0.70 | 0.04 | 1.35 |
| Other fruits | 6.82 | 5.03 | 8.61 | 8.66 | 6.88 | 10.44 |
| Dark green leafy vegetables | 24.62 | 21.38 | 27.86 | 25.90 | 22.93 | 28.88 |
| Cruciferous vegetables | 1.36 | 0.38 | 2.35 | 1.52 | 0.61 | 2.43 |
| Deep orange vegetables | 11.63 | 9.28 | 13.98 | 11.86 | 9.70 | 14.03 |
| Other vegetables | 84.88 | 82.37 | 87.39 | 81.24 | 78.79 | 83.69 |
| Legumes | 28.44 | 25.11 | 31.77 | 28.61 | 25.58 | 31.64 |
| Deep orange tubers | <0.01 | <0.01 | 0.43 | 0.04 | <0.01 | 0.49 |
| Nuts and seeds | 1.04 | 0.14 | 1.94 | 0.86 | 0.17 | 1.56 |
| Whole grains | 22.76 | 19.61 | 25.91 | 20.98 | 18.23 | 23.72 |
| Liquid oils | 98.85 | 98.07 | 99.64 | 98.73 | 97.91 | 99.55 |
| Fish and shellfish | 45.91 | 42.27 | 49.54 | 43.47 | 40.26 | 46.69 |
| Poultry and game meat | 4.20 | 2.67 | 5.73 | 4.56 | 3.16 | 5.96 |
| Low-fat dairy | <0.01 | <0.01 | 0.43 | 0.04 | <0.01 | 0.49 |
| Eggs | 8.90 | 6.75 | 11.04 | 7.92 | 6.15 | 9.69 |
| High-fat dairy | 8.68 | 6.74 | 10.62 | 7.59 | 5.91 | 9.28 |
| Red meat | 4.64 | 2.97 | 6.31 | 4.23 | 2.75 | 5.71 |

## Table A9: Control Group Probabilities of Medium or High Consumption by Healthy GDQS Food Group and Gender at Baseline and Endline from Farm Household Sample (ANGeL)

| **Baseline (2016)** | | | | | | |
| --- | --- | --- | --- | --- | --- | --- |
|  | **Men** | | | **Women** | | |
| Food Group | Prob. High or Med Cons. | 95% CI | | Prob. High or Med Cons. | 95% CI | |
| Citrus fruits | 1.53 | 0.69 | 2.37 | 1.79 | 0.87 | 28.88 |
| Deep orange fruits | 0.19 | <0.01 | 0.69 | 0.23 | <0.01 | 0.78 |
| Other fruits | 6.22 | 4.50 | 7.93 | 6.59 | 4.99 | 8.18 |
| Dark green leafy vegetables | 36.05 | 32.67 | 39.44 | 34.69 | 31.39 | 37.99 |
| Cruciferous vegetables | 31.22 | 27.91 | 34.52 | 30.84 | 27.62 | 34.07 |
| Deep orange vegetables | 1.83 | 0.79 | 2.86 | 1.92 | 0.75 | 3.09 |
| Other vegetables | 93.57 | 91.85 | 95.29 | 93.41 | 91.74 | 95.08 |
| Legumes | 27.79 | 24.60 | 30.99 | 27.26 | 24.11 | 30.41 |
| Deep orange tubers | 0.04 | <0.01 | 0.51 | 0.10 | <0.01 | 0.63 |
| Nuts and seeds | 0.56 | <0.01 | 1.21 | 0.71 | <0.01 | 1.42 |
| Whole grains | 26.60 | 23.30 | 29.91 | 23.81 | 20.71 | 26.92 |
| Liquid oils | 99.59 | 99.00 | 100.19 | 99.29 | 98.67 | 99.90 |
| Fish and shellfish | 73.70 | 70.57 | 76.83 | 74.22 | 71.15 | 77.29 |
| Poultry and game meat | 12.24 | 9.89 | 14.59 | 10.91 | 8.66 | 13.16 |
| Low-fat dairy | 0.04 | <0.01 | 0.51 | <0.01 | <0.01 | 0.42 |
| Eggs | 17.97 | 15.37 | 20.57 | 16.04 | 13.54 | 18.55 |
| High-fat dairy | 11.72 | 9.55 | 13.89 | 10.44 | 8.46 | 12.42 |
| Red meat | 7.85 | 5.80 | 9.90 | 7.06 | 5.21 | 8.91 |
| **Endline (2018)** | | | | | | |
|  | **Men** | | | **Women** | | |
| Food Group | Prob. High or Med Cons. | 95% CI | | Prob. High or Med Cons. | 95% CI | |
| Citrus fruits | 1.65 | 0.69 | 2.62 | 1.05 | 0.25 | 1.84 |
| Deep orange fruits | 0.11 | <0.01 | 0.60 | <0.01 | <0.01 | 0.49 |
| Other fruits | 5.79 | 4.21 | 7.38 | 10.13 | 8.17 | 12.09 |
| Dark green leafy vegetables | 24.27 | 21.01 | 27.53 | 24.53 | 21.49 | 28.88 |
| Cruciferous vegetables | 29.37 | 25.94 | 32.81 | 28.71 | 25.44 | 31.98 |
| Deep orange vegetables | 5.60 | 3.66 | 7.54 | 5.71 | 3.92 | 7.51 |
| Other vegetables | 95.40 | 93.77 | 97.02 | 93.68 | 91.98 | 95.38 |
| Legumes | 28.12 | 24.87 | 31.38 | 27.97 | 24.87 | 31.07 |
| Deep orange tubers | 0.11 | <0.01 | 0.60 | <0.01 | <0.01 | 0.39 |
| Nuts and seeds | 0.40 | <0.01 | 0.98 | 0.31 | <0.01 | 0.90 |
| Whole grains | 28.12 | 24.77 | 31.47 | 26.33 | 23.21 | 29.46 |
| Liquid oils | 99.73 | 99.11 | 100.35 | 99.41 | 98.79 | 100.03 |
| Fish and shellfish | 72.78 | 69.46 | 76.10 | 73.55 | 70.42 | 76.68 |
| Poultry and game meat | 13.78 | 11.14 | 16.43 | 14.22 | 11.64 | 16.80 |
| Low-fat dairy | 0.21 | <0.01 | 0.73 | 0.07 | <0.01 | 0.64 |
| Eggs | 18.31 | 15.52 | 21.09 | 16.43 | 13.85 | 19.01 |
| High-fat dairy | 11.28 | 9.14 | 13.42 | 10.30 | 8.33 | 12.26 |
| Red meat | 4.06 | 2.47 | 5.65 | 4.24 | 2.65 | 5.83 |

## Table A10: Gender Differences in Dietary Indicators, Alternative Estimation Model (Household Covariates, Village FE, and Village clustered SE)

|  | Ultra-Poor Households | | | | | Farm Households | | | | | |
| --- | --- | --- | --- | --- | --- | --- | --- | --- | --- | --- | --- |
| **Variable** | **Caloric Intake** | **CAR** | **DDS** | **GDQS** | **Prob. of Adequate Consumption** | **Caloric Intake** | **CAR** | **DDS** | **GDQS** | **Prob. of Adequate Consumption** |  |
|  |  |  |  |  |  |  |  |  |  |  |  |
| Women | -431.53 *** | -0.0098* | -0.15*** | -0.49*** | -0.74*** | -303.35 *** | 0.10*** | -0.088*** | -0.30*** | -0.46*** |  |
| Age at Baseline (ln) | -138.71 *** | 0.053*** | 0.18*** | -0.0063 | -0.24* | -206.66*** | 0.0092 | 0.13*** | -0.086* | -0.087 |  |
| Household Size (ln) | -133.97*** | 0.018 | 0.23*** | 0.70*** | 2.46*** | -203.81 *** | 0.022** | 0.14*** | 1.49*** | 3.29*** |  |
| Education Level of Household Head |  |  |  |  |  |  |  |  |  |  |  |
| Completed Pre-school | 45.39 | 0.024 | 0.10 | 0.28 | -0.17 | 41.26 | 0.013 | 0.29* | 1.47*** | 3.86*** |  |
| Completed some or all primary school | 33.79 | 0.024** | 0.036 | 0.091 | 0.26 | 12.45 | 0.0090 | 0.090** | 0.36*** | 0.63*** |  |
| Completed some or all secondary or post-secondary school | 20.72 | 0.030* | 0.096 | 0.30* | 0.68* | -32.89*** | 0.0034 | 0.11*** | 0.28*** | 0.55** |  |
| Household owns land | 47.64* | 0.010 | 0.060 | 0.098 | 0.46* | 29.15 | 0.015 | -0.0076 | 0.062 | 0.27 |  |
| Female-headed household | 18.23 | 0.031* | 0.090 | 0.38* | 0.45 | -52.42* | -0.037* | 0.048 | -0.11 | 0.15 |  |
| Per capita monthly expenditures, Taka(ln) | 345.98*** | 0.15*** | 0.88*** | 1.64*** | 4.93*** | 194.02*** | 0.12*** | 0.82*** | 2.31*** | 5.77*** |  |
| Constant | 925.90*** | -0.39*** | -3.49*** | -7.06*** | -20.51*** | 2015.37*** | -0.18** | -2.85*** | -12.74*** | -27.24*** |  |
| Number of Observations | 12970 | 12970 | 12970 | 12970 | 12970 | 12854 | 12854 | 12854 | 12854 | 12854 |  |
| Adj. R-squared | 0.14 | 0.042 | 0.075 | 0.053 | 0.082 | 0.084 | 0.063 | 0.097 | 0.13 | 0.15 |  |

## Table A11: Reported Consumption of Food Outside the Home, 24HR versus 7DDR Surveys (Ultra-Poor Households)

|  | 24HR | 7DDR |
| --- | --- | --- |
| **Food item** | Mean gram/day | Mean gram/day |
| Rice/Jao | 4.77 | 0.16 |
| Panta Bhaat | 1.78 | 10.21 |
| Khichuri | 5.90 | 1.78 |
| Polao/Biryani/Tehari | 1.13 | 0.016 |
| Ruti/Parota | 7.48 | 0.028 |
| Bonroti/paoroti | 8.06 | 66.09 |
| Paes/firni/cooked firni | 1.28 | 0.14 |
| Pitha | 1.44 | 0.30 |
| Halua | 0.79 | 1.33 |
| Bharta | 1.30 | 0.54 |
| Bhaji | 3.96 | 0.016 |
| Jhol curry | 8.97 | 0.058 |
| Bhuna curry | 4.16 | 0.0034 |
| Daal | 8.08 | 0 |
| Salad | 0.11 | 0 |
| Sweets | 3.89 | 0.0036 |
| Curd | 0.28 | 0.049 |
| Alur chap | 0.094 | 0.00042 |
| Singara | 1.92 | 0.0113 |
| Puri | 0.97 | 21.94 |
| Piaju | 0.39 | 6.13 |
| Chhola/ghugni/boot | 0.14 | 1.02 |
| Chanachur | 2.21 | 0.32 |
| Biscuit | 17.28 | 0.86 |
| Cake | 4.18 | 0.05 |
| Patties | 0.09 | 0 |
| Chips | 0.59 | 0.056 |
| Gaja | 0.61 | 0.21 |
| Murali | 0.60 | 0.03 |
| Nimki | 0.04 | 0.18 |
| Any fried food | 1.42 | 0 |
| Any boiled food | 0.52 | 0 |
| Pickles/Chutney | 0.20 | 0 |
| Total | 94.63 | 111.54 |
| Observations | 164736 | 161643 |

Notes: TMRI survey data was used to compare the reported household consumption of food outside the home between the 24HR survey (reported by the female in-charge of preparing and serving meals) and the 7DDR (reported by the male household head).

# Dietary Indicator Scores, by Household Member Type

# Table A12: Differences Dietary Indicators in Dietary by Household Member Type

|  | Ultra-Poor Households | | | Farm Households | | |
| --- | --- | --- | --- | --- | --- | --- |
| **Variable** | Caloric Intake | CAR | DDS | Caloric Intake | CAR | DDS |
|  |  |  |  |  |  |  |
| Female | -385.88*** | -0.030*** | -0.14*** | -217.69*** | 0.10*** | -0.074*** |
|  |  |  |  |  |  |  |
| Household Member Type |  |  |  |  |  |  |
| Child (2-9y) | -833.75*** | 0.030* | -0.32*** | -879.38*** | 0.004 | -0.31*** |
| Adolescent (10-18y) | -382.61*** | -0.016 | -0.18*** | -280.71*** | -0.010 | -0.078* |
| Elder | -454.93*** | 0.011 | -0.13*** | -427.02*** | -0.038*** | -0.12*** |
|  |  |  |  |  |  |  |
| Gender-Age Group Interaction |  |  |  |  |  |  |
| Female#Child (2-9y) | 336.18*** | 0.075*** | 0.093*** | 149.71*** | -0.078*** | 0.015 |
| Female#Adolescent (10-18y) | 243.93*** | 0.098*** | 0.072** | 19.082 | -0.051*** | 0.000 |
| Female#Elder | -89.75*** | 0.050*** | -0.060* | -118.06*** | 0.024* | -0.035 |
|  |  |  |  |  |  |  |
| Age at Baseline (ln) | 328.61*** | 0.050*** | 0.30*** | 262.65*** | 0.048*** | 0.24*** |
| Constant | 1653.34*** | 0.76*** | 2.89*** | 1667.61*** | 0.70*** | 3.73*** |
| Number of Observations | 19591 | 19591 | 19591 | 16982 | 16982 | 16982 |
| Adj. R-squared | 0.611 | 0.031 | 0.253 | 0.554 | 0.105 | 0.171 |

Notes: * p <0.10; **p <0.05; ***p<0.001. The reference-level for household member type is adult, and male#adult for the interaction effect. The reference-level for education of household head is “no schooling”. Models include household fixed effects and household-clustered standard errors.

## Figure A7: Caloric Intake by Household Member Type in Ultra-Poor Households, OLS Predicted Values

## Figure A8: Caloric Intake by Household Member Type in Farm Households, OLS Predicted Values

## Figure A9: CAR by Household Member Type in Ultra-Poor Households, OLS Predicted Values

## Figure A10: CAR by Household Member Type in Farm Households, OLS Predicted Values

## Figure A11: DDS by Household Member Type in Ultra-Poor Households, OLS Predicted Values

## Figure A12: DDS by Household Member Type in Farm Households, OLS Predicted Values

# Additional Tests

### Figure A7: Probability of Medium or High Consumption of Unhealthy Food Groups in Ultra-Poor Households, LPM Predicted Values

## Figure A8: Probability of Medium or High Consumption of Unhealthy Food Groups in Farm Households, LPM Predicted Values

# 5. Further Information on Dietary Measurement Methods

### Table A13: Estimated Energy Requirements for Adolescents and Adults based on Physical Activity Level (1,2)

| Sex | Age range in completed years (inclusive) | Average weight (kg) | Low Activity – Total Energy Expenditure in kcal | Moderate Activity – Total Energy Expenditure in kcal | High Activity – Total Energy Expenditure in kcal |
| --- | --- | --- | --- | --- | --- |
| Female | 15 | 46.3 | 1970.6 | 2318.4 | 2666.1 |
|  | 16 | 47.4 | 1990.0 | 2341.2 | 2692.4 |
|  | 17 | 48.1 | 2001.9 | 2355.2 | 2708.4 |
|  | 18 | 48.6 | 2010.1 | 2364.8 | 2719.5 |
|  | 19-29 | 48.9 | 1876.4 | 2239.6 | 2602.7 |
|  | 30-59 | 48.9 | 1926.1 | 2298.8 | 2671.6 |
|  | 60+ | 48.9 | 1708.4 | 2039.0 | 2369.7 |
| Male | 15 | 50.6 | 2432.9 | 2862.3 | 3291.6 |
|  | 16 | 53.7 | 2522.3 | 2967.4 | 3412.5 |
|  | 17 | 56.0 | 2585.7 | 3042.0 | 3498.3 |
|  | 18 | 57.6 | 2631.2 | 3095.6 | 3559.9 |
|  | 19-29 | 58.4 | 2435.2 | 2906.6 | 3377.9 |
|  | 30-59 | 58.4 | 2391.3 | 2854.1 | 3317.0 |
|  | 60+ | 58.4 | 1970.6 | 2352.0 | 2733.4 |

### Table A14: Adjustments in Energy Requirements for Pregnancy and Lactation (1–3)

| Reason for adjustment | Age/time range | Additional Energy Expenditure in kcal |
| --- | --- | --- |
| Lactation | Child less than 6 months (exclusive/predominate breastfeeding) | 500 |
| Lactation | Child 6 to 11 months (continued breastfeeding) | 400 |
| Lactation | Child 12-24 months (continued breastfeeding at the same level as 6-11 months) | 400 |
| Pregnancy | First trimester | 100.6 |
| Pregnancy | Second trimester | 295.2 |
| Pregnancy | Third trimester | 441.0 |
| Pregnancy | Average (trimester not recorded) | 278.9 |

### Table A15: Estimated Energy Requirements for Children and Young Adolescents (1)

|  | Male | | Female | |
| --- | --- | --- | --- | --- |
| Age in complete years | Average weight (kg) | Moderate Activity – Total Energy Expenditure in kcal | Average weight (kg) | Moderate Activity – Total Energy Expenditure in kcal |
| 2 | 13.3 | 1117.2 | 12.7 | 1031.4 |
| 3 | 15.3 | 1231.5 | 15.0 | 1150.5 |
| 4 | 17.3 | 1340.7 | 17.2 | 1260.8 |
| 5 | 19.3 | 1447.0 | 19.1 | 1352.6 |
| 6 | 21.6 | 1568.3 | 21.1 | 1452.5 |
| 7 | 24.0 | 1694.8 | 23.5 | 1562.7 |
| 8 | 26.6 | 1825.6 | 26.4 | 1688.7 |
| 9 | 29.5 | 1965.3 | 29.8 | 1826.0 |
| 10 | 32.3 | 2097.7 | 33.2 | 1942.9 |
| 11 | 35.1 | 2226.6 | 36.1 | 2044.4 |
| 12 | 38.5 | 2380.3 | 39.3 | 2142.6 |
| 13 | 42.6 | 2555.9 | 42.2 | 2223.0 |
| 14 | 46.9 | 2723.5 | 44.6 | 2280.9 |
| 15 | 50.6 | 2862.3 | 46.3 | 2318.4 |
| 16 | 53.7 | 2967.4 | 47.4 | 2341.2 |
| 17 | 56.0 | 3042.0 | 48.1 | 2355.2 |
| 18 | 57.6 | 3095.6 | 48.6 | 2364.8 |

## Table A16: GDQS Food Groups and Scoring (6)

|  |  | **Categories of Consumed Amounts (grams/day)** | | | | **Points Assigned** | | | |
| --- | --- | --- | --- | --- | --- | --- | --- | --- | --- |
| Healthy Food Groups | Food Group | Low | Middle | High | Very High | Low | Middle | High | Very High |
|  | Citrus fruits | 24 | 24–69 | 69 |  | 0 | 1 | 2 |  |
|  | Deep orange fruits | 25 | 25–123 | 123 |  | 0 | 1 | 2 |  |
|  | Other fruits | 27 | 27–107 | 107 |  | 0 | 1 | 2 |  |
|  | Dark green leafy vegetables | 13 | 13–37 | 37 |  | 0 | 2 | 4 |  |
|  | Cruciferous vegetables | 13 | 13–36 | 36 |  | 0 | 0.25 | 0.5 |  |
|  | Deep orange vegetables | 9 | 9–45 | 45 |  | 0 | 0.25 | 0.5 |  |
|  | Other vegetables | 23 | 23–114 | 114 |  | 0 | 0.25 | 0.5 |  |
|  | Legumes | 9 | 9–42 | 42 |  | 0 | 2 | 4 |  |
|  | Deep orange tubers | 12 | 12–63 | 63 |  | 0 | 0.25 | 0.5 |  |
|  | Nuts and seeds | 7 | 7–13 | 13 |  | 0 | 2 | 4 |  |
|  | Whole grains | 8 | 8–13 | 13 |  | 0 | 1 | 2 |  |
|  | Liquid oils | 2 | 2–7.5 | 7.5 |  | 0 | 1 | 2 |  |
|  | Fish and shellfish | 14 | 14–71 | 71 |  | 0 | 1 | 2 |  |
|  | Poultry and game meat | 16 | 16–44 | 44 |  | 0 | 1 | 2 |  |
|  | Low-fat dairy | 33 | 33–132 | 132 |  | 0 | 1 | 2 |  |
|  | Eggs | 6 | 6–32 | 32 |  | 0 | 1 | 2 |  |
| Healthy food groups when consumed in moderation | High-fat dairy (in milk equivalents) | 35 | 35–142 | 142-734 | >734 | 0 | 1 | 2 | 0 |
|  | Red meat | 9 | 9–46 | 46 |  | 0 | 1 | 0 |  |
| Unhealthy food groups | Processed meat | 9 | 9–30 | 30 |  | 2 | 1 | 0 |  |
|  | Refined grains and baked goods | 7 | 7–33 | 33 |  | 2 | 1 | 0 |  |
|  | Sweets and ice cream | 13 | 13–37 | 37 |  | 2 | 1 | 0 |  |
|  | Sugar-sweetened beverages | 57 | 57–180 | 180 |  | 2 | 1 | 0 |  |
|  | Juice | 36 | 36–144 | 144 |  | 2 | 1 | 0 |  |
|  | White roots and tubers | 27 | 27–107 | 107 |  | 2 | 1 | 0 |  |
|  | Deep fried food | 9 | 9–45 | 45 |  | 2 | 1 | 0 |  |

### Table A17: PAL by Occupation and Gender

|  |  | **Ultra-Poor Households** | | **Farm Households** | |
| --- | --- | --- | --- | --- | --- |
|  |  | **Men** | **Women** | **Men** | **Women** |
|  |  | (N = 8273) | (N = 9493) | (N = 7481) | (N = 8449) |
| **PAL** | **Current main occupation** | 5 (0.1%) | 4 (0.1%) | 4 (0.1%) |  |
| **Light** | Apprentice | 18 (0.3%) | 11 (0.2%) |  | 1 (0.0%) |
|  | Beggar |  |  |  |  |
|  | Contractor |  |  | 12 (0.2%) |  |
|  | Doctor | 2 (0.0%) |  | 20 (0.3%) |  |
|  | Other/undisclosed |  |  |  |  |
|  | Driver of motor vehicle | 130 (2.2%) |  | 201 (3.3%) | 1 (0.0%) |
|  | Engineer |  |  |  | 1 (0.0%) |
|  | Factory worker | 15 (0.3%) | 8 (0.1%) | 12 (0.2%) | 4 (0.1%) |
|  | Fish Trader | 37 (0.6%) |  | 10 (0.2%) |  |
|  | Food processing | 1 (0.0%) | 2 (0.0%) |  |  |
|  | Government/ parastatal | 13 (0.2%) | 3 (0.0%) | 39 (0.6%) | 3 (0.0%) |
|  | Hair cutter | 35 (0.6%) |  | 10 (0.2%) | 1 (0.0%) |
|  | Handicrafts | 16 (0.3%) | 39 (0.5%) | 13 (0.2%) | 19 (0.3%) |
|  | herbal doctor/kabiraj | 4 (0.1%) |  |  |  |
|  | Housewife | 6 (0.1%) | 2498 (34.7%) | 3 (0.0%) | 4119 (60.2%) |
|  | Jobless | 32 (0.6%) | 13 (0.2%) | 50 (0.8%) | 2 (0.0%) |
|  | Large trader (large shop or wholesale) | 8 (0.1%) |  | 57 (0.9%) | 1 (0.0%) |
|  | Lawyer/deed writer/Moktar | 1 (0.0%) |  | 10 (0.2%) | 1 (0.0%) |
|  | Medium trader (shop or small store) | 142 (2.5%) | 2 (0.0%) | 381 (6.3%) | 5 (0.1%) |
|  | Midwife |  | 5 (0.1%) |  | 2 (0.0%) |
|  | NGO worker | 3 (0.1%) | 2 (0.0%) | 5 (0.1%) | 11 (0.2%) |
|  | Other salaried worker | 45 (0.8%) | 2 (0.0%) | 76 (1.3%) | 14 (0.2%) |
|  | Other self-employed | 98 (1.7%) | 37 (0.5%) | 28 (0.5%) | 5 (0.1%) |
|  | Other wage labor | 290 (5.0%) | 52 (0.7%) | 100 (1.7%) | 4 (0.1%) |
|  | Physically/mentally challenged | 157 (2.7%) | 191 (2.7%) | 122 (2.0%) | 164 (2.4%) |
|  | Private enterprise (salesperson, manager) | 57 (1.0%) | 7 (0.1%) |  |  |
|  | Private tutor/house tutor | 6 (0.1%) | 7 (0.1%) | 1 (0.0%) | 3 (0.0%) |
|  | Religious leader | 4 (0.1%) |  | 13 (0.2%) |  |
|  | Retired | 107 (1.9%) | 99 (1.4%) | 107 (1.8%) | 111 (1.6%) |
|  | Service (private sector ) |  |  | 128 (2.1%) | 15 (0.2%) |
|  | Small industry |  | 3 (0.0%) | 2 (0.0%) |  |
|  | Small trader (roadside stand or stall) | 285 (4.9%) | 22 (0.3%) | 198 (3.3%) | 5 (0.1%) |
|  | Student | 207 (3.6%) | 201 (2.8%) | 368 (6.1%) | 285 (4.2%) |
|  | Tailor/seamstress | 20 (0.3%) | 40 (0.6%) | 24 (0.4%) | 61 (0.9%) |
|  | Tea garden worker | 1 (0.0%) | 1 (0.0%) |  | 1 (0.0%) |
|  | Teacher | 5 (0.1%) |  | 44 (0.06%) | 18 (0.02%) |
|  | Transport worker (bus/truck helper) | 25 (0.4%) |  | 16 (0.3%) |  |
|  | Veterinary/paravet doctor | 1 (0.0%) | 1 (0.0%) | 2 (0.0%) | 1 (0.0%) |
|  | Village doctor | 7 (0.1%) |  |  |  |
| **Moderate** | Animal Breeder |  | 1 (0.0%) |  | 2 (0.0%) |
|  | Blacksmith | 1 (0.0%) | 1 (0.0%) | 4 (0.1%) |  |
|  | Carpenter | 105 (1.8%) |  | 88 (1.5%) |  |
|  | Commercially feed producer | 1 (0.0%) |  | 1 (0.0%) | 1 (0.0%) |
|  | Fisherman | 288 (5.0%) | 10 (0.1%) | 9 (0.1%) | 1 (0.0%) |
|  | House maid |  | 73 (1.0%) | 6 (0.1%) | 6 (0.1%) |
|  | Potter | 3 (0.1%) |  |  |  |
| **Heavy** | Agricultural day labor | 1457 (25.2%) | 174 (2.4%) | 413 (6.9%) | 37 (0.5%) |
|  | Clothes washer | 1 (0.0%) |  | 1 (0.0%) |  |
|  | Cobbler | 11 (0.2%) |  | 183 (3.0%) |  |
|  | Construction labor | 76 (1.3%) | 5 (0.1%) | 24 (0.4%) |  |
|  | Earth work (government program) | 8 (0.1%) | 14 (0.2%) | 32 (0.5%) | 29 (0.4%) |
|  | Earth work (other) | 314 (5.4%) | 12 (0.2%) | 32 (0.5%) |  |
|  | Electrician | 7 (0.1%) |  | 23 (0.4%) |  |
|  | Goldsmith/silversmith | 2 (0.0%) |  | 7 (0.1%) |  |
|  | Homestead farming | 3 (0.1%) | 20 (0.3%) | 1 (0.0%) | 4 (0.1%) |
|  | Mason | 98 (1.7%) |  |  |  |
|  | Mechanic (vehicles) | 11 (0.2%) |  | 9 (0.1%) | 1 (0.0%) |
|  | Milk collector/Milk seller | 4 (0.1%) |  | 2 (0.0%) |  |
|  | Milk producer |  | 1 (0.0%) |  |  |
|  | Plumber | 9 (0.2%) |  | 5 (0.1%) |  |
|  | Porter | 27 (0.5%) |  | 2 (0.0%) | 1 (0.0%) |
|  | Raising fish / fishpond | 20 (0.3%) | 1 (0.0%) | 15 (0.2%) |  |
|  | Raising livestock | 81 (1.4%) | 1540 (21.4%) | 75 (1.2%) | 638 (9.3%) |
|  | Raising poultry | 2 (0.0%) | 2042 (28.4%) | 7 (0.1%) | 1157 (16.9%) |
|  | Repairman (appliances) | 15 (0.3%) |  | 9 (0.1%) |  |
|  | Rickshaw/van pulling | 541 (9.4%) |  | 121 (2.0%) |  |
|  | Scavenger | 1 (0.0%) |  |  | 1 (0.0%) |
|  | Sharecropper/tenant | 474 (8.2%) | 14 (0.2%) | 997 (16.6%) | 24 (0.4%) |
|  | Sweeper | 3 (0.1%) | 4 (0.1%) | 1 (0.0%) | 1 (0.0%) |
|  | Working own farm (crop) | 430 (7.4%) | 10 (0.1%) | 1892 (31.4%) | 76 (1.1%) |

# 6. References

1. Waid JL, Bogard JR, Thilsted SH, Gabrysch S. Estimates of average energy requirements in Bangladesh: Adult Male Equivalent values for use in analyzing household consumption and expenditure surveys. Data Brief [Internet] Elsevier Inc.; 2017 [cited 2021 Jun 9];14:101–6. Available from: http://creativecommons.org/licenses/by/4.0/

2. Food and Agriculture Organization of the United Nations. Human energy requirements. Report of a Joint FAO/WHO/UNU Expert Consultation. Rome; 2004.

3. Picciano MF. Pregnancy and Lactation: Physiological Adjustments, Nutritional Requirements and the Role of Dietary Supplements. J Nutr [Internet] Oxford Academic; 2003 [cited 2022 May 14];133:1997S-2002S. Available from: https://academic.oup.com/jn/article/133/6/1997S/4688112

4. Choudhury S, Nahar Q, Faruque O, Siddiquee MA. Desirable Dietary Pattern for Bangladesh. 2013.

5. FAO/WHO/UNU. Energy and protein requirements:  Report of  a  joint  FAO/WHO/UNU  expert consultation. WHO Technical Report Series No. 724. 1985.

6. Intake–Center for Dietary Assessment. The Global Diet Quality Score: Data Collection Options and Tabulation Guidelines. Washington, DC; 2021.
